# Primary Gastric Adenocarcinoma with Thyroid Transcription Factor-1 Positivity Mimicking Gastric Metastasis from Lung Cancer: A Case Report

**DOI:** 10.70352/scrj.cr.25-0677

**Published:** 2026-01-31

**Authors:** Yoshihito Iijima, Masahito Ishikawa, Shun Iwai, Akihiro Shioya, Nozomu Motono, Sohsuke Yamada, Hidetaka Uramoto

**Affiliations:** 1 Department of Thoracic Surgery, Kanazawa Medical University, Kahoku-gun, Ishikawa, Japan; 2 Department of Pathology and Laboratory Medicine, Kanazawa Medical University, Kahoku-gun, Ishikawa, Japan

**Keywords:** thyroid transcription factor-1, primary gastric adenocarcinoma, neoplasm metastasis, immunohistochemistry, diagnostic challenge

## Abstract

**INTRODUCTION:**

Thyroid transcription factor-1 (TTF-1) is a well-established immunohistochemical marker for tumors of lung and thyroid origin. Metastatic adenocarcinomas are often tested for TTF-1 expression to identify their primary site. Herein, we report a rare case of TTF-1–positive primary gastric adenocarcinoma that was initially misdiagnosed and treated as postoperative gastric metastasis from primary lung cancer.

**CASE PRESENTATION:**

A 58-year-old man underwent thoracoscopic right upper lobectomy with systemic lymph node dissection for lung cancer of the right upper lobe. The pathological diagnosis was invasive adenocarcinoma (pT2bN0M0, Stage IIA). He received 3 courses of postoperative adjuvant platinum doublet chemotherapy. One year and 2 months after surgery, he was diagnosed with gastric and adrenal tumors. Immunohistochemical analysis of the gastric lesion demonstrated TTF-1 positivity, leading to the diagnosis of gastric and adrenal metastatic recurrence of lung cancer. The patient received triple therapy with carboplatin, pemetrexed, and pembrolizumab, followed by maintenance therapy with pemetrexed and pembrolizumab. During treatment, the adrenal metastasis achieved a complete response; however, the gastric lesion showed gradual progression on endoscopic follow-up. As primary gastric cancer could not be ruled out, the patient underwent robot-assisted distal gastrectomy with D2 lymph node dissection and Billroth I reconstruction, 3 years and 4 months following lung resection. Immunohistochemical staining of the gastric tumor revealed adenocarcinoma that was positive for TTF-1 and caudal-related homeodomain protein 2 (CDX2) and negative for napsin A. In contrast, lung cancer tissue was weakly positive for TTF-1 and negative for napsin A and CDX2. Based on the immunohistochemical staining and histological findings, the final diagnosis was primary gastric adenocarcinoma. The postoperative course was uneventful, and maintenance chemotherapy with pemetrexed and pembrolizumab was resumed. Four years and 10 months after cancer surgery, the patient remains in complete response.

**CONCLUSIONS:**

This case highlights the diagnostic challenge posed by TTF-1–positive gastric tumors, which may be mistaken for metastatic lesions from lung cancer. As TTF-1 expression is not entirely specific to tissues of lung or thyroid origin, diagnosis should be based on a comprehensive evaluation of morphological and immunohistochemical findings, together with clinical information, including treatment response and disease course.

## Abbreviations

CBDCA
: carboplatin
CK
: cytokeratin
HER2
: human epidermal growth factor receptor-related 2
IHC
: immunohistochemistry
LND
: lymph node dissection
PD-L1
: programmed death-ligand 1
PEM
: pemetrexed
TTF-1
: thyroid transcription factor-1

## INTRODUCTION

Thyroid transcription factor-1 (TTF-1) is a homeodomain-containing DNA-binding protein initially identified in thyroid follicular epithelial cells and subsequently in type II pneumocytes and Clara cells of the lung.^1,2)^ TTF-1 is widely used as an immunohistochemical marker for tumors of lung and thyroid origin.^1–3)^ In tissue analysis, immunohistochemistry (IHC) with anti-TTF-1 antibodies demonstrates nuclear staining in these normal cell types as well as in their corresponding neoplastic cells. In lung carcinoma, TTF-1 expression is positive in small cell carcinomas and adenocarcinomas, but negative in squamous cell carcinomas.^1)^ Although TTF-1 expression is generally considered specific to the thyroid and lungs, immunopositive staining has also been reported in colonic adenocarcinomas, ovarian epithelial neoplasms, and uterine tumors.^2)^ Metastatic adenocarcinomas are often tested for TTF-1 expression to identify their tissue of origin.^3,4)^ Herein, we report a rare case of TTF-1–positive primary gastric adenocarcinoma that was initially diagnosed and treated as postoperative gastric metastasis from primary lung cancer.

## CASE PRESENTATION

**Fig. 1:**
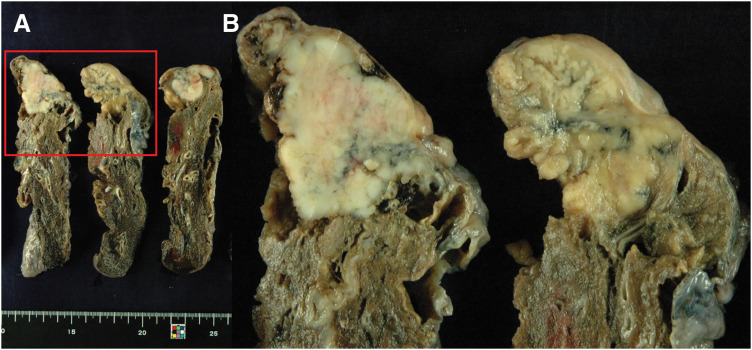
Macroscopic findings of lung cancer. Macroscopically, a solid, grayish-white mass measuring 42 × 32 mm with a right-leaning septum was observed. The red rectangles in (**A**) are within the same range as those in (**B**).

**Fig. 2:**
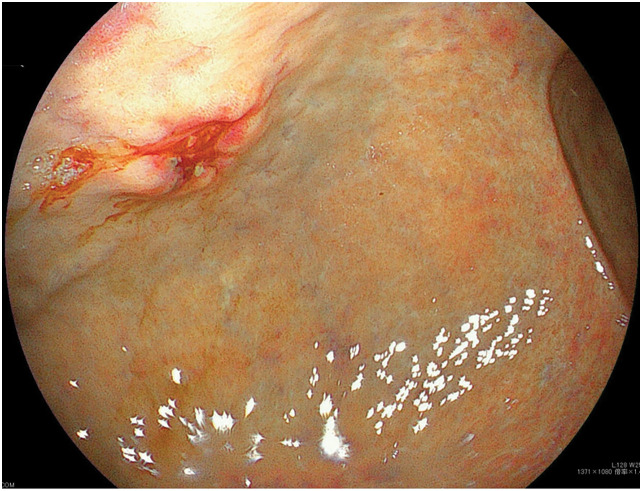
Upper gastrointestinal endoscopic findings. A raised lesion with an irregular central ulcer was observed on the anterior wall of the mid-gastric body. The submucosal tumor appeared exposed in the lumen.

**Fig. 3:**
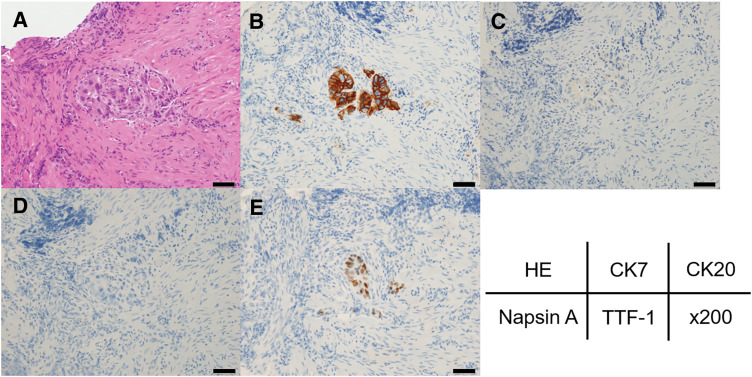
H–E and Immunohistochemistry staining of endoscopic gastric tumor biopsies. (**A**) H–E staining showed atypical cells infiltrating the submucosa and muscularis mucosa. Duct formation was observed along with diffuse and cord-like proliferation. Immunohistochemical staining revealed that the tumor cells were (**B**) weakly positive for CK7 and (**C**) negative for CK20. (**D**) Napsin A was negative, and (**E**) TTF-1 was focally positive, leading to the diagnosis of gastric metastasis of lung cancer. Magnification, ×200. The length of the black bar is 50 μm. CK 7, cytokeratin 7; H–E, hematoxylin–eosin; TTF-1, thyroid transcription factor-1

A 58-year-old man underwent thoracoscopic right upper lobectomy with systemic lymph node dissection (LND) for lung cancer of the right upper lobe. The pathological diagnosis was invasive adenocarcinoma (pT2bN0M0, Stage IIA; **Fig. 1**). He received 3 courses of cisplatin and docetaxel combination chemotherapy as postoperative adjuvant treatment and subsequently underwent upper gastrointestinal endoscopy during a medical checkup. Upper gastrointestinal endoscopy revealed a raised lesion with an irregular central ulcer on the anterior wall of the mid-gastric body (**Fig. 2**). The submucosal tumor appeared exposed in the lumen. The endoscopic findings were more suggestive of metastatic gastric tumors than of primary gastric cancer. A PET-CT scan revealed a right adrenal tumor. No locoregional recurrence of the lung cancer was observed. Hematoxylin and eosin staining of the gastric tumor revealed atypical cells infiltrating the submucosa and muscularis mucosa. Duct formation was observed, along with diffuse and cord-like proliferation (**Fig. 3A**). Immunohistochemical staining revealed that the tumor cells were weakly positive for cytokeratin 7 (CK7) and negative for CK20. Napsin A was negative, and TTF-1 was focally positive (**Fig. 3B**–**3E**). Based on the immunohistochemical profile, the morphological features of a gastric submucosal tumor, and the presence of adrenal metastasis on PET-CT, a diagnosis of gastric metastasis from lung cancer was established, rather than that of a primary gastric cancer.

Lung cancer tissues were analyzed for programmed death-ligand 1 (PD-L1) and other biomarkers via next-generation sequencing, and high PD-L1 expression was confirmed at 55%. The patient received 4 cycles of triple therapy with carboplatin (CBDCA), pemetrexed (PEM), and pembrolizumab, followed by 31 cycles of maintenance therapy with PEM and pembrolizumab.

During this period, the adrenal metastasis achieved a complete response, whereas endoscopic follow-up showed slow progression of the gastric tumor. At this point, the primary lung tumor was under control, and no distant metastasis was detected elsewhere in the body. The gastric tumor formed an ulcer, creating the risk of bleeding if chemotherapy was continued. Furthermore, the possibility of primary gastric cancer could not be ruled out. Therefore, after consultation with the Gastrointestinal Surgery Department, the decision to proceed with resection was made. The patient underwent robot-assisted distal gastrectomy, LND (D2), and Billroth I reconstruction 3 years and 4 months after lung resection.

**Fig. 4:**
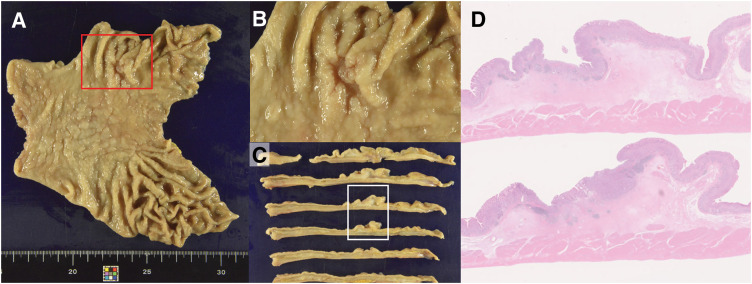
Macroscopic and whole slide image findings of gastric cancer. (**A**–**C**) Macroscopically, a 15 × 9-mm 0-IIc lesion was observed 36 mm from the rostral stump and 70 mm from the anal stump. The red rectangle in (**A**) is within the same range as that in (**B**). A whole slide image revealed adenocarcinoma with irregular tubular shapes, fusion, and branching. Some vesicular nest-like growth was observed. The white rectangle in (**C**) is within the same range as that in (**D**).

**Fig. 5:**
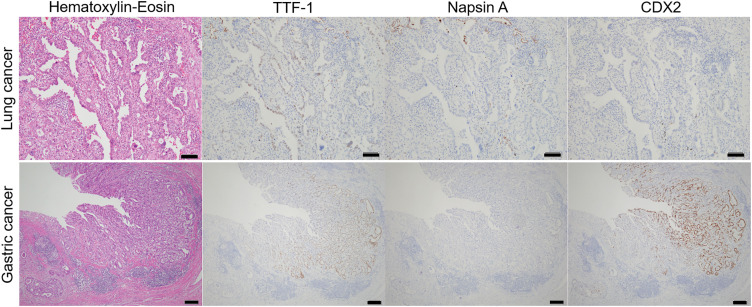
Comparison of H–E and IHC staining of lung cancer and gastric cancer. From the top left to right: H–E staining, TTF-1, napsin A, and CDX2 staining of lung cancer. Magnification, ×100. The length of the black bar is 100 μm. Bottom row, from left to right: H–E staining, TTF-1, napsin A, and CDX2 staining of gastric cancer. Magnification, ×40. The length of the black bar is 200 μm. CDX2, caudal-related homeodomain protein 2; H–E, hematoxylin–eosin; IHC, immunohistochemistry; TTF-1, thyroid transcription factor-1

Macroscopically, a 15 × 9-mm 0-IIc lesion was observed 36 mm from the rostral stump and 70 mm from the anal stump (**Fig. 4A**–**4C**). Histologically, the tumor was an adenocarcinoma showing irregular tubular and partial alveolar proliferation with fusion and branching (**Fig. 4D**). Immunohistochemical staining was positive for TTF-1 and negative for napsin A, consistent with the immunostaining results for the gastric biopsy specimens. Notably, caudal-related homeodomain protein 2 (CDX2) was also positive. In contrast, lung cancer tissue was weakly positive for TTF-1 and negative for napsin A and CDX2 (**Fig. 5**). Based on the immunohistochemical staining and histological findings, the final diagnosis was gastric adenocarcinoma (pT1b2N1M0, Stage IB). No adverse events were observed postoperatively, and maintenance chemotherapy with PEM and pembrolizumab was resumed. Four years and 10 months following lung cancer surgery, the patient remains in complete response.

## DISCUSSION

Hematogenous metastases to the stomach remain rare despite its rich blood supply, with a postmortem incidence of 0.2%–9%.^5)^ In most studies, breast, malignant melanoma, and lung cancers are the most common malignancies that metastasize to the stomach.^5)^ In addition to the esophagus, metastasis to the gastrointestinal tract from lung cancer is uncommon and often asymptomatic, with an incidence of 0.3%–1.77%.^6–8)^ Hematogenous metastases to the stomach are usually submucosal.^5,9)^ The macroscopic appearance of these submucosal gastric metastases has been radiographically described as a bull’s-eye sign and endoscopically as volcano-like or umbilicated on the tip.^5,9)^ These lesions are found more commonly in the upper and middle thirds of the stomach, although there are several reported cases of metastases in the lower third and even the pylorus.^5,9)^ In this case, a raised lesion with an irregular central ulcer was observed endoscopically on the anterior wall of the mid-gastric body. The submucosal tumor appeared exposed in the lumen. The endoscopic findings were more suggestive of metastatic gastric tumors than of primary gastric cancer.

TTF-1–positive gastric cancer is also rare, with previous reports indicating a prevalence of 0.5%–0.9%.^1,3)^ The reason for aberrant staining of the transcription factor TTF-1 in gastrointestinal tumors, however, has not yet been determined. Fukagawa et al. demonstrated that NK2 homeobox 1 (NKX2-1)/TTF-1, a known master regulator of lung differentiation, can regulate downstream target genes in both AGS and NUGC4 cells.^10)^ NKX2-1/TTF-1 is ectopically expressed in fundic gland–type (GA-FG) gastric adenocarcinoma and can regulate downstream genes in GA-FG tumors, suggesting that it plays an essential role in GA-FG development.

In routine pathology diagnostics, the 2 most commonly applied TTF-1 clones are the SPT24 clone and 8G7G1/1. Evidence indicates that the SPT24 clone shows higher sensitivity and less erratic cytoplasmic staining than the 8G7G1/1 clone.^1)^ However, the SPT24 clone has been reported to be positive in at least 25% of gastric adenocarcinoma cases.^3)^ Choi et al. attributed this discrepancy to differences in the amount of tumor tissue available for analysis between tissue microarrays, which provided a more limited view of the tumor and paraffin-embedded tissue blocks.^3)^ TTF-1 IHC, particularly with the presumably more sensitive SPT24 clone, should be employed with the caveat that expression is not specific to tissues of lung and thyroid origin.^3)^ Furthermore, it was concluded that reliance on morphological, clinical, and additional immunohistochemical data is essential for accurate classification of tumors of unknown origin.^3)^ Möller et al. analyzed a tissue microarray containing 17772 specimens obtained from 152 different types of tumors.^11)^ Their comparative analysis of TTF-1 and napsin A showed a sensitivity and specificity of 94.1% and 86.1% (TTF-1-positive), 87.4% and 97.8% (napsin A-positive), and 84.9% and 99.1% (TTF-1- and napsin A-positive) in distinguishing lung adenocarcinoma, respectively.^11)^ That study included 415 analyzable gastric cancer specimens for TTF-1 IHC: 167 diffuse-type, 188 intestinal-type, and 60 mixed-type. The TTF-1 positivity rates were 0% for the diffuse type, 5.9% for the intestinal type, and 5% for the mixed type, respectively. Möller et al. concluded that TTF-1 is a sensitive but insufficiently specific marker for lung adenocarcinoma, and that a small proportion of TTF-1-positive gastrointestinal adenocarcinomas mimics enteric-type lung adenocarcinoma, suggesting that integrated analysis of TTF-1 and napsin A may improve the diagnostic specificity of lung adenocarcinoma.^11)^ Co-expression of TTF-1 and napsin A is reported only in 0.5% of gastric carcinomas; thus, the rate of tumor misinterpretation may potentially be decreased when using both antibodies to distinguish gastric carcinomas from lung adenocarcinomas.^1)^ Noack et al. proposed using “gastrointestinal immunohistochemical markers,” such as CK20 and CDX2, to solve the diagnostic dilemma of distinguishing primary lung adenocarcinoma from metastatic gastrointestinal tumors.^1)^ However, while additional immunohistochemical staining has shown utility in proving gastrointestinal origin, clinical evidence is still required.^1)^ In this case, although we were unable to obtain information on the antibodies used, the gastric tumor was TTF-1-positive, and recurrent adrenal metastasis of lung cancer was also noted at the same time; therefore, the overall diagnosis was gastric metastasis of lung cancer.

Based on the results of the KEYNOTE-189^12)^ and other trials,^13)^ platinum combination therapy with a PD-1/PD-L1 inhibitor, along with immune checkpoint inhibitor monotherapy, is strongly recommended (recommendation level 1B) as the first-line treatment in Japan for patients with a performance status of 0–1 and PD-L1 expression of ≥50%. In contrast, based on the results of the KEYNOTE-062 trial,^14)^ pembrolizumab is recommended in combination with chemotherapy as the first-line treatment for human epidermal growth factor receptor-related 2 (HER2)-negative, unresectable, advanced or recurrent gastric cancer.^15)^ In this case, after the diagnosis of recurrence, 4 courses of triple therapy with CBDCA, PEM, and pembrolizumab were administered, followed by 31 courses of maintenance therapy with PEM and pembrolizumab. During this time, the adrenal metastasis achieved a complete response, whereas endoscopic evaluation indicated slow growth of the gastric tumor. This aberrant response to treatment led us to suspect gastric cancer. Gastric cancer has not recurred, and HER2 testing has not been performed; however, the administration of pembrolizumab for recurrent lung cancer might have slowed the progression of gastric cancer.

## CONCLUSIONS

We encountered a case of TTF-1–positive gastric cancer that was difficult to differentiate from metastatic gastric tumors. Although TTF-1 has high sensitivity depending on the antibody used, it should be used considering that its expression is not specific to the lung or thyroid tissues. Therefore, reliance on morphological and pathological data, as well as on treatment history, such as response to chemotherapy, is essential for correctly classifying tumors of unknown etiology.

## DECLARATIONS

### Availability of data and materials

All data generated or analyzed during this study are included in this published article.
